# Can Brain Natriuretic Peptides and Osteoprotegerin Serve As Biochemical Markers for the Detection of Aortic Pathology in Children and Adolescents with Turner Syndrome?

**DOI:** 10.3389/fendo.2017.00142

**Published:** 2017-07-04

**Authors:** Meenal Mavinkurve, Clodagh S. O’Gorman

**Affiliations:** ^1^Department of Paediatrics, Clinical School, International Medical University, Seremban, Malaysia; ^2^Department of Paediatrics, Graduate Entry Medical School, University of Limerick, Limerick, Ireland; ^3^Department of Paediatric Endocrinology and Diabetes, University Hospital Limerick, Limerick, Ireland

**Keywords:** Turner syndrome, vasculopathy, B-type natriuretic peptide, N-terminal pro BNP, osteoprotegrin

## Abstract

Turner syndrome (TS) is a chromosomal disorder that affects 1:2,000 females. It results from either the complete or partial loss of the X chromosome as well as other aberrations. Clinical features of TS include short stature, delayed puberty, and congenital cardiac malformations. TS children also have an increased prevalence of cardiometabolic risk factors, which predisposes them to complications like coronary artery disease, cerebrovascular-related deaths, and aortic dissection. Early cardiac imaging, such as echocardiography and cardiac magnetic resonance imaging, are recommended to detect underlying aortic pathology. However, these modalities are limited by cost, accessibility, and are operator dependent. In view of these shortcomings, alternative methods, like vascular biomarkers, are currently being explored. There are only a few studies that have examined the relationship between B-type natriuretic peptide (BNP), N-terminal pro BNP (NT pro-BNP), and osteoprotegerin (OPG) and aortic disease in TS, and thus the data are only in proof-of-concept stages. Further meticulous longitudinal studies are required before BNP, NT pro-BNP, and OPG are used as vascular biomarkers for the detection of aortic disease in childhood and adolescent TS.

## Introduction

Turner syndrome (TS) is the commonest chromosomal abnormality in females affecting approximately 50 in 100,000 live female births (1). TS commonly results from either the complete or partial loss of a single X chromosome and though the phenotype varies with the type of chromosomal abnormality, it is hallmarked by short stature and ovarian failure (2, 3). Left-sided cardiac malformations such as co-arctation of the aorta and bicuspid aortic valves (BAV) are seen in 50% of TS patients (4, 5). The presence of BAV in TS is associated with an increased incidence of aortic dissection (Ao D) such that 95% of TS cases who develop Ao D have underlying BAV. Hypertension, overweight, glucose intolerance, and vascular endothelial dysfunction also contribute to the pathogenesis of Ao D (6–21). Children with TS have abnormal aortic distensibility and carotid intima media thickness which are present even in the absence of cardiometabolic risk factors. Furthermore, the functional changes in the vascular endothelium are evident prior to the structural changes in children with TS (22, 23).

Guidelines recommend regular cardiac surveillance with echocardiography and cardiac MRI as part of the structured care of young children with TS. It is aimed in detecting early aortic valve disease and any underlying adverse cardiometabolic risk factors in an attempt to reduce the risk of fatal Ao D (3). However, it is likely that these modes may not be sensitive enough to detect the early vasculopathic changes that are inherent to TS, given that functional endothelial changes precede structural changes.

Soluble vascular biomarkers (SVBs) may offer viable alternatives to assessing vascular health in TS. SVBs are measureable substances, which serve as indicators of clinical end points. They can be used to monitor and predict response to therapy. Some SVBs have clinical utility in the diagnosis of certain aortic and cardiac conditions in adults. The American Heart Association lists nine criteria that SVBs should fulfill before they can be considered as clinical tools (Table 1) (24). Some biomarkers, such as low-density lipid cholesterol, B-type natriuretic peptide (BNP), and atrial natriuretic peptide, fulfill these criteria and they are currently used in clinical practice. Similarly, there are SVBs that are associated with vasculopathy. These include BNP, N-terminal pro BNP (NT pro-BNP), and osteoprotegerin (OPG). On the premise that TS is an inherently vasculopathic condition, an intriguing question that rises is whether these biomarkers have a potential role to detect subclinical aortic disease, monitor treatment response, and predict outcome childhood TS associated with early aortic disease (25, 26). This review will discuss data from studies on B type natriuretic peptide (BNP), NT pro-BNP, and OPG in childhood TS. Table 2 summarizes studies on biochemical markers in aortic pathology in TS.

**Table 1:** AHA criteria for soluble vascular biomarkers.

AHA biomarker criteria
1. Proof of concept
*Is the biomarker different in subjects with and without the clinical outcome?*
2. Prospective validation
*Does the biomarker predict the development of future outcomes?*
3. Incremental value
*Does the biomarker add predictive information over current risk markers?*
4. Clinical utility
*Does the biomarker predict risk sufficiently to guide management?*
5. Clinical outcomes
*Does the use of the biomarker improve clinical outcomes?*
6. Cost-effectiveness
*Does the use of the biomarker justify its additional costs?*
7. Ease of use
*Is the biomarker widely applicable?*
8. Methodological consensus
*Can the biomarker be used to make comparisons between studies?*
9. Reference values
*Are there established reference ranges?*

**Table 2:** Summary of studies examining B-type natriuretic peptide (BNP), N-terminal pro BNP (NT pro-BNP), and osteoprotegerin (OPG) in Turner syndrome (TS) patients.

Reference	Biomarker	Population	Study design	Main findings
Gravholt et al. (11)	NT pro-BNP Renin Aldosterone	TS (*n* = 9) Mean age 29.7 ± 5.6 years Controls (*n* = 8)	Randomized placebo controlled cross over study	– NT pro-BNP levels were higher in the TS cohort. – Renin and aldosterone levels were comparable between the 2 groups. – Treatment with hormone replacement therapy (HRT) did not influence the levels of NT pro-BNP.

Gutin et al. (35)	NT pro-BNP	TS (*n* = 114) Controls (*n* = 27) Age 18–67 years (mean age 37.4 ± 12 years)	Cross-sectional	– NT pro-BNP levels are significantly different between the 2 groups. – NT pro-BNP levels are significantly higher in the TS group after excluding TS patients with dilated aorta. – Highly significant correlation between NT pro-BNP levels and descending aortic diameter. – TS participants with dilated ascending aorta had significantly high mean NT pro-BNP levels as compared to those who did not have dilated ascending aorta.

Uçar et al. (36)	BNP ANP hsCRP PRA IGF1 IGFBP3	TS (*n* = 61) Age 6.6–21.3 years (mean age 12.6 years) Controls (*n* = 61)	Cross-sectional	– TS cohort and healthy controls were matched for age, sex, 24-h ambulatory BP, Nocturnal BP dipping. – TS cohort had significantly high cIMT, β-index, Einc SDS values. – TS cohorts had significantly higher levels of BNP, atrial natriuretic peptide (ANP), hsCRP even after correcting for BMI and puberty. – BNP maintained a significant positive correlation with all measures of arterial stiffness (cIMT, Einc, β-index, and Distensibility coefficient). – ANP only retained a positive correlation with cIMT and hsCRP had a positive correlation with β-index, Einc, and distensibility coefficient.

Buzi et al. (46)	OPG RANKL	Controls (*n* = 46) Age 1–14 years (Mean 7.8 years SD 3.76) TS (*n* = 10) Mean age 10.8 years No estrogen replacement On human GH therapy	Cross-sectional	– OPG levels in normal children (aged 1–14 years) were highest in infancy and decreased with increasing age. – No correlation between OPG levels and BMI, height, weight, puberty. – OPG levels in TS cohort were lower than normal in age and sex-matched controls. – No difference in RANKL levels between TS and controls.

Trolle et al. (47)	OPG	Controls (*n* = 68) TS (*n* = 99) Age matched	Prospective	– TS patients were significantly different from healthy controls in terms of BMI, BSA, blood pressure measurements. – OPG levels were significantly lower in the TS cohort at baseline and at follow-up and at the end of the study. – Lower levels of OPG were more pronounced in the Monosomy X compared to mosaics, but not significant. – OPG levels correlated with BSA-indexed distal descending aortic diameter at all 3 time points.

## B Type Natriuretic Peptides

B type natriuretic peptides and NT pro-BNP are neurohormones that are encoded by the BNP gene, NPBB. These neurohormones are released in response to cardiac muscle stretch, ventricular ischemia, or volume overload and mediate their effects through the renin–angiotensin–aldosterone system act to lower systemic blood pressure. BNP is noted to be elevated in clinical conditions such as congestive cardiac failure, hypertension, and coronary artery disease in adults (27). BNP levels and NT pro-BNP levels also correlate with the clinical severity of aortic stenosis (AS), predict the need for aortic valve replacement in AS, and predict mortality associated with AS (28–33). BNP can be sampled easily in the clinical setting with the use of commercially available kits ensuring timely measurements for diagnosis and therapeutic monitoring (34). However, interpretation of BNP levels should be done with caution using sex and age appropriate reference ranges and also take into account BMI and glomerular filtration rate as these factors influence circulating BNP and NT pro-BNP levels. BNP and NT pro-BNP appear as promising SVBs in non-TS-related cardiac disease and aortic disease in non-TS patients. Thus, presenting a logical starting point from which to assess if there are sufficient data supporting BNP and NT pro-BNP as potential biomarkers in childhood TS.

## B Natriuretic Peptides and TS

The relationship between NT pro-BNP and TS was first investigated by Gravholt et al. (11). The primary aim of this study in nine TS women, all karyotype confirmed and aged between 29.7 ± 5.6 years, was to determine the impact of hormone replacement therapy (HRT) on sympathovagal tone in TS patients. B natriuretic peptide levels, which were quantified in both the TS patients and healthy peers, were noted to be elevated in all TS subjects but particularly in the subset of TS subjects who had nocturnal hypertension. None of the participants had an ECHO during the study, so it is difficult to ascertain whether the TS cohort with nocturnal hypertension had underlying aortic disease, even though they all reportedly had normal systolic function on an ECHO done prior to the study (11). Interestingly, over the 2 years while on HRT, an increase in the NT pro-BNP levels was not observed in the TS group, suggesting that NT pro-BNP levels are not influenced by HRT and so may be of value in TS children undergoing pubertal induction. Despite the lack of a discernible connection between NT pro-BNP and aortic disease in this TS cohort, the study did report the novel finding that NT pro-BNP levels are distinctly abnormal in TS individuals, thus posing the question for future studies regarding the utility of NT pro-BNP as a potential biomarker in TS.

Another study by Gutin et al. (35) examined the relationship between NT pro-BNP and aortic disease in 114 karyotype confirmed *normotensive* adult TS patients. This study demonstrated that NT pro-BNP levels were elevated in TS subjects as compared to healthy controls and that the NT pro-BNP levels were high irrespective of the presence of underlying aortic disease, confirming the findings from Gravholt et al. (11), but further elucidating that high NT pro-BNP levels are intrinsic to TS in the absence of underlying aortic disease and clinically detectable hypertension. A sub-analysis of the TS group with aortic dilatation demonstrated a strong positive correlation between NT pro-BNP levels and the diameter of the ascending aorta as well as BSA-indexed ascending aortic dilatation confirming that there is indeed a relationship between NT pro-BNP levels and radiologically detectable aortic disease in TS women.

Uçar et al. (36), more recently, examined the association between BNP and arterial stiffness in *young* normotensive TS patients. Sixty-one TS subjects, with a mean age of 12.6 years, normal cardiac anatomy, and matched for systolic and diastolic BP SD scores, were compared with healthy controls on measures of arterial stiffness and BNP levels. The TS cohort, the youngest participant being 6.6 years old, had significantly higher measures of arterial stiffness than healthy peers, such as cIMT, β-index, distensibility coefficient, and incremental elastic modulus (Einc). Furthermore, sub-analyses revealed a robust correlation between BNP levels and *all* measures of arterial stiffness: cIMT, β-index, Einc, and distensibility coefficient. This study reports several important findings: first, that BNP levels can be assessed in very young TS children; second, that BNP levels are increased in young TS individuals who do not have clinical hypertension, suggesting the idea that elevated BNP levels in young TS patients may signify an inherent vasculopathy; third, that BNP levels were not influenced by BMI nor puberty, making it a potentially useful tool in childhood, a period during which BMI is fluctuating and pubertal induction is ongoing.

## OPG in TS

Osteoprotegerin is a glycoprotein from the tumor necrosis factor family of cytokines, which is linked to several cardiac conditions. OPG functions as a decoy receptor for receptor activator of nuclear factor kb ligand (RANKL) and thus blocks the interaction between RANKL and NFkB. NFkB activation is a key regulator of inflammatory, vascular, and skeletal gene transcription pathways. OPG levels are increased in advanced atherosclerosis and abdominal aortic aneurysm, silent myocardial infarction, unstable angina, and heart failure (37–42). OPG levels can also predict outcome and mortality in individuals with coronary artery disease (42–44). These data suggest that circulating OPG levels can be quantified in disease states and that they are distinctly abnormal in *adult* cardiovascular disease.

In children, OPG levels have been quantified in specific cohorts, such as type 1 diabetes mellitus (T1DM) and chronic renal failure. Fekih et al. (45) quantified circulating OPG levels in a childhood T1DM cohort (*n* = 143; mean age 12 years) with cardiometabolic risk factors such as prolonged duration (≥4 years) of diabetes, HbA1c ≥ 7%, dyslipidemia, and microalbuminuria (≥30 mg/24 h). Their study revealed that OPG levels were significantly higher (*p* < 0.0001) in the T1DM cohort. OPG levels also correlated significantly with several cardiovascular risk factors, in particular, OPG levels were significantly increased in children who had more than three cardiovascular risk factors. Though this study is not specific to a TS cohort, it nonetheless demonstrates several important findings that can be applied to future TS cohorts. First, it shows that OPG is quantifiable in children and that normative ranges exist. Second, it highlights that OPG levels are influenced by cardiometabolic risk factors, which are prevalent in the TS childhood population also (7). Finally, given that OPG levels are significantly raised in T1DM children with more cardiometabolic risk factors, raising the question of whether elevated OPG levels can identify those TIDM children who are likely to have a poor prognosis. This question is equally applicable to a TS childhood cohort, given their underlying cardiometabolic risk factor profile.

The relationship between OPG and TS is only being examined more recently. The first, a cross-sectional study by Buzi et al. (46) examined the difference between OPG levels in healthy controls, *children* with precocious puberty, rheumatoid arthritis, and TS aged between 1 and 14 years. Interestingly, they showed that the TS cohort had significantly *lower* OPG levels than age-matched controls but superimposable to the cohort with precocious puberty. It is difficult to draw definitive conclusions from this study about the value of OPG as a biomarker in TS as neither the cardiac nor the metabolic bone health profile of the TS cohort was reported. Nonetheless, it is the first study to demonstrate that OPG levels in the least are deranged in childhood TS.

Trolle et al. reported on a cohort of 99 *adult* TS subjects over a 5-year period (47) with the aim of establishing any differences between OPG and aortic pathology in TS. They corroborated the finding by Buzi et al. that TS individuals have significantly *lower* levels of OPG levels as compared to healthy volunteers, but they also added to the previous study by conducting aortic measurements. They showed that there was a positive correlation between OPG levels and aortic diameter, in particular those individuals with aortic dilatation. Together, these findings confirm that OPG levels are abnormal in adult TS cohorts, in particular those who have underlying aortic dilatation. However, contrary to the study by Fekih et al. (45), OPG levels did not correlate with traditional cardiometabolic factors like hypertension, hsCRP, cholesterol levels, or nocturnal hypertension, all of which are associated with TS. Nonetheless, it suggests that OPG levels are a biomarker worth examining in *childhood* TS in relation to cardiometabolic risk factors and aortic disease.

## BNP, NT pro-BNP, and OPG as SVBs in Childhood TS

Several studies have reported on the association between TS and the levels of certain biomarkers, namely BNP, NT pro-BNP, and OPG. However, only two of these studies specifically examine childhood TS cohorts, thus highlighting the paucity of data on this topic. Given the dearth of information in childhood TS and vascular biomarkers, conclusions about their usefulness as clinical tools are difficult to assert. Despite the lack of studies in this field, several conclusions can still be drawn on which future studies should be based. First, these studies demonstrate that certain vascular biomarkers are indeed abnormal in TS patients and that they do fulfill some of the criteria of SVBs as set out by the AHA (Table 2). The studies, though few in number, also demonstrate that the biomarker levels are deranged in childhood TS before the onset of aortic complications. This highlights the importance of investigating further with respect to their usefulness as predictive and prognostic tools. In order to determine the predictive ability of BNP, NT pro-BNP, and OPG for aortic disease, prospective longitudinal studies are needed. Most of the studies discussed in this review are cross-sectional studies and so are unable to draw conclusions on whether these SVBs are useful in predicting aortic disease in TS. Quantifying SVB levels in childhood TS in conjunction with cardiac imaging and measures of endothelial function could give valuable information on the diagnostic ability of these biomarkers and whether they can serve as complementary tools to traditional cardiac imaging techniques. Furthermore, though normative values exist for BNP, NT pro-BNP, and OPG, and it is known that certain variables, such as BMI or puberty, can influence these values, which are relevant to the TS cohort as are variables like growth hormone therapy and pubertal induction (48). Hence, the impact of these common therapies used in TS on SVB levels need to be elucidated. Future studies should also take the opportunity to examine whether these SVBs can be used to monitor aortic disease progression and response to conventional medical and surgical therapy for aortic disease. None of the studies have examined the cost-effectiveness of SVBs in diagnosing TS-related aortic disease. This needs to be determined before they are widely adopted as tools in clinical practice, in addition to determining whether their widespread use can positively impact on aortic disease management and outcomes in childhood TS. Finally, none of the studies have attempted to determine the physiological mechanisms underpinning the aberrations in SVB levels in childhood TS. Elucidating these mechanisms may assist in developing new targeted therapies for the management of aortic disease in childhood TS.

## Conclusion

The utility of SVBs, in particular BNP, NT pro-BNP, and OPG, in childhood TS to predict aortic pathology is limited. Data from studies examining these SVBs in adult TS cohorts support the fact that their levels are distinct in the TS cohort, in particular those with underlying aortic pathology. At the present time, the data are insufficient to make the claim that these SVBs can be used as clinical tool. Nonetheless, this dearth of information presents numerous opportunities for future research which should focus on consolidating them as biomarkers as per the AHA criteria and also by determining their usefulness as clinical tools for diagnosis, predicting outcomes, and prognosis.

## Author Contributions

MM contributed to the idea of the article and conducted the literature search and drafting the manuscript. CO contributed to idea of the article, the drafting of the manuscript, and assisted in editing the manuscript prior to final submission.

## Conflict of Interest Statement

The authors declare that the research was conducted in the absence of any commercial or financial relationships that could be construed as a potential conflict of interest.
